# High prevalence of multi drug resistant tuberculosis in people living with HIV in Western India

**DOI:** 10.1186/s12879-019-4042-z

**Published:** 2019-05-08

**Authors:** Neil Saldanha, Kiran Runwal, Charulata Ghanekar, Sunil Gaikwad, Shrivallabh Sane, Sanjay Pujari

**Affiliations:** 1 Consultant Biostatistician, Poona Hospital and Research Center, Pune, India; 2 Institute of Infectious Diseases, Pune, India

**Keywords:** MDRTB, HIV, India, Prevalence

## Abstract

**Background:**

Most studies assessing drug resistant tuberculosis (DRTB) in human immunodeficiency virus (HIV) co-infected patients in India have used conventional culture- based systems to diagnose DRTB that have a longer turnaround time leading to risk of amplification of resistance to an empirical regimen. We determined the prevalence of DRTB amongst people living with HIV (PLHIV) using the line probe assay and determined risk factors associated with the presence of multi drug resistant tuberculosis (MDRTB).

**Methods:**

A Cross-sectional study was undertaken at Poona Hospital and Research Center (PHRC) and the Institute of Infectious Diseases, two tertiary level private care centers in Pune, India. Consenting PLHIV with confirmed Pulmonary TB (PTB) and/or extra-pulmonary TB (EPTB) diagnosed based on detection of Mycobacterium TB by line probe assay (Geno Type MTBDRplus version 2) on clinical specimens were included. Those with documented past history of DRTB were excluded. Resistance against anti-TB drugs was determined by the same assay. The prevalence of any form of drug resistant TB (DRTB), MDRTB, Rifampicin resistant TB (RRTB) and Isoniazid (INH) mono-resistant TB were determined as the proportion of these amongst all included PLHIV-TB. A multivariate analysis was conducted to determine risk factors that were statistically associated with MDRTB, DRTB, RRTB and INH mono-resistant TB.

**Results:**

Two hundred PLHIV were recruited. The prevalence (95% CI) of MDRTB, INH mono- resistance and RR resistance was 12.5% (7.9–17.1%), 9% (6.9–11.2%) and 2.5% (1.4–3.6%), respectively. The prevalence (95% CI) of MDRTB among new and relapsed patients was 8.8% (6.5–11.1%) and 23.1% (17.2–28.9%), respectively. Tuberculosis relapse was the only factor significantly associated with MDRTB, DRTB and INH mono-resistant TB.

**Conclusion:**

We document a high prevalence of drug resistance to anti-TB drugs including MDRTB among PLHIV in our setting using Geno Type MTBDRplus directly on clinical specimens. This validates the WHO recommendation of performing routine rapid molecular resistance testing prior to initiating anti-TB treatment among all PLHIV with presumptive TB. Using rapid molecular testing especially Geno Type MTBDRplus (that detects resistance to INH and Rifampicin simultaneously) reduces the turn-around time helping in optimizing treatment.

## Background

Tuberculosis (TB) is one of the most common opportunistic infections in people living with human immunodeficiency virus (PLHIV) and a major contributor to morbidity and mortality [1, 2]. Drug-resistant TB (DRTB), including multi-drug resistant TB (MDRTB), defined as resistance to isoniazid (INH) and rifampicin (Rif), is rapidly emerging and threatens to derail the success of TB control programs in high endemic TB settings [3]. Although HIV infection by itself does not increase the risk of acquiring primary DRTB, using intermittent treatment for TB has been associated with the development of acquired rifampicin resistance [4].

According to the Global Tuberculosis Report 2018, India has the highest burden of TB (27%) and MDRTB (24%) in the world [5]. In 2017, estimated proportion of MDRTB amongst new and previously treated cases were 2.0 and 12% respectively in India.

An estimated 9% of incident TB cases in 2017 were amongst PLHIV globally. Few studies in India have explored the prevalence of DRTB among PLHIV [6, 7]. In a study amongst PLHIV attending a public sector Antiretroviral center in Mumbai, the prevalence of MDRTB (drug susceptibility testing using phenotypic liquid culture) was 38% and only previous history of TB was significantly associated with the diagnosis of DRTB. An MDRTB rate of 5.6% was reported amongst PLHIV from South India, however Geno Type MTBDRplus for diagnosis was done retrospectively on culture isolates [7] and risk factors were not explored.

The World Health Organization (WHO) recommends baseline resistance testing prior to initiating an anti-Tuberculous treatment (ATT) for all PLHIV with active TB. The line probe assay effectively detects INH and Rif resistance rapidly and has an advantage over the Xpert TB/Rif that detects only Rif resistance [8]. Information on prevalence and risk factors for MDRTB (diagnosed by rapid molecular tests on clinical specimens) amongst PLHIV is urgently needed in India. We determined the prevalence of DR-TB among PLHIV using the line probe assay and determined risk factors associated with the presence of MDRTB.

## Methods

### Design and study site

The study employed a cross-sectional design and was conducted between Jan 2014 and Dec 2016. Two tertiary level care centers, Poona Hospital and Research Center (PHRC) and the Institute of Infectious Diseases (IID) in Pune, Western India participated. Both these centers are urban and situated in the same city. While IID is an out-patient clinic providing comprehensive care to PLHIV and other infectious diseases, PHRC provides in-patient and laboratory support to IID. The study protocol was approved by the Independent Ethics Committee of PHRC.

### Patients

All consenting PLHIV with confirmed pulmonary (PTB) and/or extra-pulmonary TB (EPTB) were consecutively recruited in the study. Confirmation was based on detection of mycobacterium TB (MTB) in clinical specimens by Line Probe Assay (Genotype MTBDRplus). For PTB, sputum or Bronchoscopic Alveolar lavage (BAL) were processed. Extrapulmonary TB was presumably diagnosed based on histo-pathologic evidence of classical caseating granulomas in clinical specimens like lymph nodes and confirmed with detection of MTB on Geno Type MTBDRplus. Those with a past history of documented DRTB were excluded.

### Procedures

A written consent was provided by all PLHIV participating in the study. Data collection and documentation was done using a standardized case report form by the principal investigator.

Demographic information including HIV related (duration since diagnosis, current, and past CD4 counts, and viral loads), ART (antiretroviral therapy) related (duration on ART, type of regimen, whether failing) and TB related (pulmonary/extrapulmonary, new/treatment cases) were collected from past medical records and self-report. HIV infection was diagnosed based on a positive result on 4th generation ELISA and rapid immunochromatography. Resistance to anti-TB drugs was determined by the line probe assay Geno Type MTBDRplus version 2, (HAIN Lifesciences GmbH, Hardwiesenstraße, Nehren, Germany). In this assay, the resistance to INH and Rif is determined by identifying mutations in the *INH A/Kat G* and *rpoB* genes, respectively. The plasma viral load was determined by COBAS TaqMan (Roche Molecular Systems, Branchburg, NJ), while CD4 counts were determined by FACSCount (BD Biosciences, Sane Jose, CA, USA).

We defined MDRTB as resistance to INH and Rif, RRTB as resistance to Rif only, and DRTB as either mono-resistance (RRTB or INH mon-resistance) or MDRTB. Primary TB was defined as diagnosed with TB for the first time and never treated with anti-TB drugs while TB relapse was defined as patients who completed a course of anti-TB treatment in the past.

### Statistical analysis

The prevalence of MDRTB was determined by calculating the proportion of patients with MDRTB among those with TB. The significance of differences among the demographic variables between the two groups (MDRTB versus non-MDRTB) was determined by Mann-Whitney test and chi-square test for continuous and categorical variables, respectively.

Multivariate analyses (logistic regression) were used to determine statistically significant risk factors for the presence of MDRTB, DRTB, RRTB and INH mono-resistant TB. The outcome variable was treated as dichotomous variable (MDRTB vs non-MDRTB, DRTB vs non-DRTB). The exploratory variables included age, gender, duration since HIV diagnosis, current CD4 counts, and PVL, type of TB (PTB and EPTB), primary and relapsed TB. A *p*-value of less than 0.05 was considered statistically significant.

A sample size of 200 was calculated assuming a prevalence of 15% for MDRTB amongst relapsed cases with 90% power and a p-value of 0.05. All analyses were done using SPSS version 20 software.

## Results

Two hundred patients with confirmed TB were recruited. The prevalence (95% CI) of MDRTB was 12.5% (7.9–17.1%). Prevalence of INH mono-resistance, RR and DRTB was 9.0% (6.9–11.2%) and 2.5% (1.4–3.6%), and 24.0% (20.9–27.0%) respectively. Sixty-two percent of the drug resistance to INH was mediated through KatG mutations.

The demographics for patients with TB, MDRTB, and non-MDRTB are summarized in Table 1.

**Table 1:** Demographics of PLHIV with all TB, MDRTB and non-MDRTB

	All TB (*n* = 200)	MDRTB (*n* = 25)	Non-MDRTB (*n* = 175)	*P* value (MDRTB vs Non-MDRTB)
Age, years				
Median (range)	41.5 (11–48)	43 (11–65)	40 (16–79)	0.70
Male sex, n (%)	134 (67.0)	18 (72.0)	116 (66.2)	0.56
Duration since HIV diagnosis, years				
Median (range)	5 (1–23)	4 (1–19)	5 (1–23)	0.21
CD4 counts				
Median (range)	114 (9–644)	90 (11–591)	120 (9–644)	0.04
ART status				
Naïve, n (%)	79 (39.5)	11 (44.0)	68 (38.9)	0.62
Experienced, n (%)	121 (60.5)	14 (56.0)	107 (61.1)	
TB				
Primary, n (%)	147 (73.5)	13 (52.0)	135 (77.1)	0.007
Relapsed, n (%)	53 (26.5)	12 (48.0)	40 (22.8)	

The median age (range) of all TB patients was 41.5 (11–48) years and 67% were males. The median duration (range) since HIV diagnosis was 5 [3–10] years. The median (range) current CD4 count was 114 (9–644)/mm^3^, while approximately 74.5% patients had a CD4 count less than 200/mm^3^. Sixty percent of patients were on ART (antiretroviral therapy) and 68.0% of patients were diagnosed to have EPTB. Lymphadenopathy (63.0%) constituted the major form of EPTB. Seventy-four percent of patients had primary TB. Among relpased cases 48.1% had previous PTB. Most patients (92.5%) with TB were smear positive.

The prevalence of MDRTB in PLHIV with CD4 < 200/mm^3^ was higher (14.0%) than those with CD4 > 200/mm^3^ (5.8%), although this difference was not statistically significant. However, PLHIV with MDRTB had a lower median CD4 count (90/mm^3^) as compared to non-MDRTB (120/mm^3^, *p* = 0.04). In multivariate analyses, only previously treated cases had higher odds for the presence of MDRTB (Table 2). The prevalence (95% CI) of MDRTB among new and relapsed patients was 8.8% (6.5–11.1%) and 22.6% (11.3–33.9%), respectively. The prevalence (95% CI) of INH mono-resistance in the new and relapsed patients was 7.4% (3.8–14.9%) and 13.5% (8.7–18.1%), respectively, while that of rifampicin mono-resistance was 2.7% (0.5–3.6%) and 1.9% (0.1–3.82%), respectively, and DRTB was 18.9% (12.6–25.2%) and 38.5% (25.2–51.7%), respectively. In multivariate analysis, only retreatment was significantly associated with higher odds of DRTB, MDRTB and INH mono-resistance, 2.4 (1.2–4.9), 2.5 (1.0–6.3) and 2.6 (1.2–5.5), respectively (Table 2).

**Table 2:** Multivariate analysis to explore risk factors for presence of DRTB, MDRTB, RRTB and INH mono-resistance

	DRTB OR (95% CI)	MDRTB OR (95% CI)	RRTB OR (95% CI)	INH mono-resistance OR (95% CI)
Age < 30				
Age 31–60	1.0 (0.2–4.3)	0.4 (0.1–4.5)	0.3 (0.1–2.9)	1.4 (0.3–6.0)
Age > 60	1.2 (0.3–4.4)	0.4 (0.1–3.9)	0.4 (0.1–3.3)	1.4 (0.4–5.1)
Gender	1.0 (0.5–2.1)	1.4 (0.5–3.6)	1.5 (0.6–3.7)	0.9 (0.4–1.9)
HIV-Duration < 5				
6–10 years	0.8 (0.3–2.3)	0.6 (0.2–2.4)	0.7 (0.3–2.3)	0.8 (0.2–2.4)
> 10 years	1.6 (0.5–4.7)	1.1(0.3–5.1)	1.3 (0.5–4.7)	1.5 (0.3–4.9)
CD4 counts < 50				
51–100	0.6 (0.2–1.8)	0.2 (0.1–1.2)	0.3 (0.1–1.2)	0.6 (0.2–1.8)
> 100	0.7 (0.3–1.6)	0.5 (0.1–1.2)	0.5 (0.1–1.5)	0.7 (0.3–1.7)
ART status	1.0 (0.5–2.4)	1.1 (0.4–3.2)	1.2 (0.5–3.1)	1.0 (0.4–2.4)
Type of TB: EPTB	0.6 (0.3–1.3)	0.5 (0.1–1.2)	0.6 (0.2–1.4)	0.6 (0.3–1.2)
Primary and relapse	2.4 (1.2–4.9)*	2.5 (1.0–6.3)**	2.18 (1.0–5.1)	2.6 (1.2–5.5)***

*p = 0.018, **p = 0.048, *** *p* = 0.012

The outcome variable was treated as dichotomous variable (MDRTB vs non-MDRTB, DRTB vs non-DRTB, RRTB vs non-RRTB and INH mono-resistant TB vs non-INH mono-resistant TB)

Odds Ratio (OR)

## Discussion

We have documented a high prevalence of overall drug resistance against anti-TB drugs including MDRTB in PLHIV. This is higher than the WHO’s global estimate of 3.5 and 20.5% MDRTB among new and retreatment cases respectively, among all TB cases irrespective of HIV infection status [9]. However, the prevalence of MDRTB in this study is similar to those documented by a few other studies in India. For instance, a study on PLHIV attending public ART program in Mumbai documented a MDRTB prevalence of 11.4% amongst new TB cases and 36.4% amongst re-treated TB cases [6]. However, the study was predominantly conducted among PLHIV belonging to lower socio-economic strata and resistance was determined using phenotypic liquid culture. We did not formally assess the socio-economic status of our patients. However, our centers are private care setups and patients accessing care pay out of pocket (middle to higher socio-economic status) unlike the public setup of the Mumbai study that provides diagnosis and treatment free. In another study from a TB control program clinic in Delhi, an MDRTB prevalence of 13.2% was documented using phenotypic methods [10].

A prevalence of MDRTB of 7%, using the line probe assay on culture isolates, was documented in a study from South India [11]. The sample size in this study was small, only 76 mycobacterial strains obtained from 67 patients. Prevalence of Rif resistant TB was 11.2%, by Xpert MTB/RIF, amongst PLHIV with TB (*n* = 867) participating in a large demonstration study across public clinics in India [12]. To the best of our knowledge, this is the largest study determining the prevalence of DRTB including MDRTB using line probe assay on clinical specimens in PLHIV in India. This provides a more robust estimate of the burden of DRTB/MDRTB in PLHIV, although it is valid only for those availing medical care in the private sector.

It is crucial to identify DRTB/MDRTB rapidly to improve clinical outcomes and prevent further transmission to the contacts. Traditional culture and susceptibility methods have a long turnaround time (up to 4–6 weeks) that delays the initiation of appropriate anti-TB regimen [13]. The turnaround time for rapid molecular tests varies from two hours to two days facilitating early treatment and preventing further transmission thus having a potential to have a major public health impact [12]. The performance of these tests has been comparable to the traditional culture and phenotypic susceptibility methods [14–16]. We did not simultaneously perform culture and susceptibility on our specimens and hence cannot comment on the accuracy of line probe assay. Historically, other studies from India have documented a higher prevalence of DRTB with phenotypic methods. Smear gradation appears to influence the probability of obtaining an interpretable result and the sensitivity of the line probe assay, indicating a significant association between the bacillary load and the test performance [14]. Heteroresistance may also explain this discordance, although this is unlikely to be true in our study.

We found no HIV related factors associated with higher odds for the presence of MDRTB in our study on multivariate analysis. However, on univariate analysis lower median CD4 count was associated with presence of MDRTB. There is conflicting evidence on HIV infection as a risk factor for the presence or development of MDRTB. In a Bayesian modeling study, HIV co-infection did not significantly affect the transmissibility and the mutation rate of MTB in patients and was not associated with the increased emergence of resistance [17].

Most of the studies have documented a higher prevalence of MDRTB in the retreated cases in PLHIV as well as those without HIV infection [6, 18]. Even in our study, the only risk factor associated with presence of MDRTB was TB relapse. Various factors may contribute to the higher risk of MDRTB in the retreated patients. Exposure to sub-optimal anti-TB regimens during previous treatment due to poor compliance, improper prescriptions, and drug storage and quality, may significantly contribute to the emergence of DRTB [19]. Apart from this, the patients may be treated with irrational fixed-dose combinations not adjusted to appropriate body weight. Intermittent TB treatment as used in the program has been associated with a higher risk of acquired rifampicin resistance, especially among PLHIV. Among PLHIV receiving thrice weekly anti-TB regimen, a higher incidence of acquired rifampicin resistance has been documented in Chennai, India [4]. While ART was associated with the lower presence of MDRTB in this study, it did not eliminate this risk completely. Finally, many previously treated patients used standard first-line WHO regimen without baseline resistance testing. In the context of undiagnosed INH resistance, further amplification of resistance to other drugs especially rifampicin may occur [20–22].

There are a few limitations of our study. We did not recruit concurrent HIV negative controls for demonstrating differences in the prevalence between the two groups as we did not have access to HIV negative patients with TB. We did not simultaneously perform phenotypic testing for detecting resistance and neither performed Genotype MTB DR plus/sl to detect resistance to ethambutol, fluoroquinolones, and aminoglycosides. Finally, our patients were belonging to the middle to high socio-economic strata and accessed care in the private health sector thus limiting external validity of our study.

## Conclusion

We have documented a high prevalence of DRTB/MDRTB in PLHIV with the line probe assay. This validates the WHO recommendation of performing routine rapid molecular resistance testing prior to initiating an anti-TB treatment in all PLHIV. These tests can also be performed on smear negative and directly on other extra-pulmonary specimens. Further studies are needed to confirm these findings, especially among PLHIV with TB accessing care in the public programs in India.

## Abbreviations

ATT: Anti-Tuberculous treatment
BAL: Bronchoscopic Alveolar lavage
DRTB: Drug resistant tuberculosis
EPTB: Extra-pulmonary TB
HIV: Human Immunodeficiency Virus
IID: Institute of Infectious Diseases
INH: Isoniazid
MDRTB: Multi drug resistant tuberculosis
MTB: *Mycobacterium tuberculosis*
PHRC: Poona Hospital and Research Center
PLHIV: People living with HIV
PTB: Pulmonary TB
Rif: Rifampicin
RRTB: Rifampicin resistant TB
TB: Tuberculosis
WHO: World Health Organization
